# The median effective doses of propofol combined with two different doses of nalbuphine for adult patients during painless gastroscopy

**DOI:** 10.3389/fphar.2022.1014486

**Published:** 2022-09-20

**Authors:** Lili Tang, Chenxuan Ye, Nan Wang, Chen Chen, Sirui Chen, Shan Gao, Xuesheng Liu

**Affiliations:** ^1^ Department of Anesthesiology, The First Affiliated Hospital of Anhui Medical University, Hefei, China; ^2^ Department of Biomedical Engineering, City University of Hong Kong, Kowloon Tong, Hong Kong SAR, China; ^3^ Department of Pharmacology, Basic Medical College, Anhui Medical University, Hefei, China

**Keywords:** nalbuphine, propofol, gastroscopy, dose-effect relationship, ED50

## Abstract

**Objective:** Propofol is the most widely administered intravenous anesthetic to induce deep sedation for gastroscopy procedures. Coadministration of nalbuphine can provide analgesia and sedation to patients experiencing visceral pain, thereby decreasing the amount of propofol needed and reducing the risk of propofol-induced adverse events. We carried out this study to determine the median effective dose (ED50) of propofol in combination with different dosages of nalbuphine and the optimal dosage of nalbuphine during painless gastroscopy.

**Methods:** We recruited sixty-five patients aged 18–60 years who underwent elective painless gastroscopy. A total of sixty-one patients were allocated randomly to the N1 group (nalbuphine 0.1 mg/kg) or N2 group (nalbuphine 0.15 mg/kg). Three minutes after administration of nalbuphine, patients received a preset dose of propofol at 2.0 mg/kg with a dose gradient of 0.2 mg/kg according to Dixon’s “up-and-down” method. The primary outcome was the ED50 of propofol in combination with nalbuphine. Hemodynamic parameters, recovery time, pain score, and adverse events were recorded as secondary outcomes.

**Results:** The ED50 of propofol in the N2 group was significantly lower than that observed in the N1 group (*p* < 0.01). Using probit analysis, the ED50 and ED95 of propofol were 1.632 mg/kg and 2.759 mg/kg in the N1 group and 1.111 mg/kg and 2.243 mg/kg in the N2 group, respectively. The incidence of hypotension in the N2 group was lower than that in the N1 group (*p* < 0.05), and the recovery time was shorter than that of the N1 group (*p* < 0.05).

**Conclusion:** In adult patients, 0.15 mg/kg nalbuphine led to a significant reduction in the ED50 and ED95 of propofol during gastroscopy. This dose of nalbuphine also reduced the incidence of hypotension and shortened the recovery time. Therefore, nalbuphine (0.15 mg/kg) combined with propofol is a safe option for enhancing recovery after painless gastroscopy in adult patients.

**Clinical Trial Registration**: [https://www.chictr.org.cn/edit.aspx?pid=126699&htm=4], identifier [ChiCTR2100053204].

## Introduction

Gastroscopy is widely used to diagnose or treat esophageal and gastric diseases and may reduce the incidence and mortality rates of upper gastrointestinal tract cancer in areas with high cancer risk in China (Xia et al., 2021). In 2018, 22.2 million gastrointestinal endoscopies were performed in the United States, and 284,844 new cases of gastrointestinal cancer were diagnosed (Peery et al., 2022). However, gastroscopy is a painful and unbearable procedure without sedation and analgesia. Therefore, the vast majority of endoscopic examinations (>98%) were performed under sedation (Cohen et al., 2006).

Propofol sedation in gastroscopy has become a mainstay of clinical practice due to its shorter onset, faster recovery, antiemetic effect, better acceptance and higher diagnostic accuracy of the procedure (Meining et al., 2007; Wang et al., 2013; Committee et al., 2018). However, propofol alone appears to have several dose-dependent adverse effects, such as respiratory depression, hypotension, hypoxemia and injection pain (Vaessen and Knape, 2016; Yin et al., 2019; Stogiannou et al., 2018). Thus, μ-receptor opioids, such as remifentanil, fentanyl or sufentanil, can be combined with propofol to reduce propofol-related adverse events in clinical practice (Xu et al., 2008; Zhang et al., 2014; Li et al., 2016; Yin et al., 2019; Yu et al., 2019; Wang et al., 2020; Dossa et al., 2021). However, there is still a high incidence of severe hypoxemia (SPO_2_ < 90%) and hypotension when these μ-receptor opioids are combined with propofol during gastrointestinal endoscopy (Xu et al., 2008; Lin et al., 2019), which may result in longer recovery time and higher financial burden.

Nalbuphine, a κ-opioid receptor agonist and μ-opioid receptor antagonist, provides analgesia, a sedative effect, less respiratory depression, and increased patient comfort (Chestnutt, 1987; Jaffe et al., 1988; Sury and Cole, 1988). Compared to sufentanil, Sun suggested that nalbuphine provides a better analgesic effect for visceral pain and higher patient satisfaction after cesarean section (Sun et al., 2020). Nalbuphine at 0.162 mg/kg combined with propofol was reported to be effective and safe for painless gastroscopy in adults (Li et al., 2021). Our center also found that 0.1 mg/kg nalbuphine could effectively inhibit the injection pain associated with propofol and reduce the total dosage of propofol needed for gastroscopy (Wang et al., 2020). However, the minimum effective dose of propofol in combination with nalbuphine has not yet been determined. Therefore, the current study was to investigate the median effective dose (ED50) of propofol combined with different doses of nalbuphine during gastroscopy in adult patients.

## Materials and methods

The current randomized controlled trial was approved by the Ethical Committee of The First Affiliated Hospital of Anhui Medical University (China, Approval No. PJ 2021-14-15) and registered in the Chinese Clinical Trial Registry (https://www.chictr.org.cn/edit.aspx?pid=126699&htm=4, ChiCTR2100053204; 14 November 2021). All patients in our study provided written informed consent.

### Patients

We recruited patients who were scheduled for painless gastroscopy or biopsy with American Society of Anesthesiologists (ASA) physical status I or II. All patients were adults aged 18–60 y with a body mass index (BMI) between 18 and 24 kg/m^2^.

The exclusion criteria were as follows: age <18 y or >60 y; ASA III or higher; overweight (body mass index >24 kg/m^2^); allergy to nalbuphine or propofol; liver or kidney dysfunction; history of neurologic, respiratory or heart diseases; mental illness, sedative or analgesic drug abuse; duration of gastroscopy >30 min; inability to provide informed consent.

### Clinical protocol

Based on the dose of nalbuphine (Rui Jing, Yichang Humanwell Pharmaceutical, Hubei, China; lot no. 21J04021), patients were assigned randomly to an N1 group (0.1 mg/kg nalbuphine) or an N2 group (0.15 mg/kg nalbuphine) at a 1:1 ratio using computer-generated randomized numbers.

All patients fasted for 8 h, had no water for 2 h and did not receive any preoperative medication before the gastroscopy. Standard physiological monitoring, including oxygen saturation (SpO_2_), blood pressure, respiratory rate (RR) and electrocardiogram, was applied every 2 min, and venous access to the upper limb was secured in the operating room. Nalbuphine in both groups was diluted into 10-ml syringe by an anesthesiologist who did not participate in the case collection. When nalbuphine was given intravenously, oxygen was supplied by a mask (5 L/min). Approximately 3 min after nalbuphine administration, propofol (AstraZeneca, Cambridge, United Kingdom; lot no. RX455) was injected within 60 s. Sedation levels were assessed with the Modified Observer’s Assessment of Alertness/Sedation Scale (MOAA/S) every minute during gastroscopy (score of 5: responds quickly to name spoken in normal tone; score of 4: lethargic response to name spoken in normal tone; score of 3: responds only after name is called loudly and/or repeatedly; score of 2: responds only after mild prodding or shaking; score of 1: responds only after painful trapezius squeeze; 0 score: no response after painful trapezius squeeze). Gastroscopy was performed after the patient’s MOAA/S score was ≤2 (Patel et al., 2005; Kang et al., 2021). All anesthetic injections and management were performed by the same senior anesthesiologist, and the examination was conducted by the same group of experienced endoscopists.

We assessed the threshold for all-or-none responses to gastroscopy using the up-and-down method (Dixon, 1991). Propofol was administered at a preselected dose of 2.0 mg/kg, and a booster injection of 0.5 mg/kg propofol was administered if the patient could not tolerate the operation of the gastroscope, which was indicated by frowning, cough, or any physical movement when gastroscopy was placed within 5 min after propofol injection. Accordingly, the dosage of propofol for the next patient was increased by a step size of 0.2 mg/kg, and if the gastroscopic examination was successfully completed, the dose for the next patient to be examined was decreased by 0.2 mg/kg. The corresponding propofol dose at the midpoint of negative and positive responses was defined as the effective dose of propofol for 1 crossover, and the effective dose in each group was the average of the 7 crossovers in this group.

The primary outcome was the ED50 of propofol in the two groups. Secondary outcomes included initial dose of propofol; duration of procedure (the time from endoscopic implantation to endoscopic withdrawal); time of opening eyes (the time from losing consciousness to opening eyes); orientation recovery time (the time from losing consciousness to answer the name and location); systolic blood pressure (SBP), diastolic blood pressure (DBP), heart rate (HR) and respiratory rate (RR) before induction (T1), after induction (T2), end of gastroscopy (T3), and opening eyes (T4); adverse events; pain score at the time of anesthesia recovery by visual analog scale (VAS, 0 = painless and 10 = severe pain); and duration of stay in the postanesthesia care unit (PACU; the time from endoscopic withdrawal to Steward score of 6). An anesthesiology resident who was blinded to group assignment recorded the data.

Systolic blood pressure decreased by more than 20% compared with the preoperative baseline value, or the mean arterial pressure was less than 60, which was regarded as perioperative hypotension. Patients with intraoperative hypotension were immediately treated with phenylephrine (20 μg). Atropine (0.5 mg) was administered if patients had bradycardia (HR < 50 bpm). If hypoxemia (SpO_2_ < 95%) appeared, the lower jaw was lifted; if the SpO_2_ did not improve or continued to drop to less than 90% (severe hypoxia), pressure-assisted ventilation with a mask was performed.

### Statistical analysis

According to another article in our center, the incidence of hypotension during gastroscopy was 44% with 0.1 mg/kg nalbuphine combined with propofol (Wang et al., 2020). We assumed a 50% reduction in hypotension with 0.15 mg/kg nalbuphine, requiring 50 samples per group to achieve a power of 0.8, an α significance level of 0.05, and a loss to follow-up rate of 0.1. We also applied the 7 crossovers recommended by the up-down method of Dixon’s approach for sample size calculation (Dixon, 1991; Chen et al., 2021). All data analyses were performed by SPSS version 23.0 (IBM Inc., Armonk, NY, United States), provided by the Medical Data Processing Center of the School of Public Health of Anhui Medical University. Normally distributed continuous variables were expressed as the means ± standard deviations (SD) and compared by independent-samples *t* test. Nonnormally distributed data are presented as medians (interquartile ranges, IQR) and analyzed by the Mann–Whitney U test. Categorical variables were expressed as frequencies (%) and compared with Fischer’s exact test. The ED50 of each group was calculated as the average of 7 crossovers of the dose of propofol, and then, the ED50 values in the two groups were compared by independent-samples *t* test. We also applied the probit method (probability unit regression) to analyze the up-and-down sequences in each group and to calculate the ED50 and 95% effective dose (ED95) of propofol in the two groups. Repeated measures ANOVA was used to analyze hemodynamic and respiratory changes. A *p* value less than 0.05 was considered statistically significant.

## Results

### Included patient information

**FIGURE 1:**
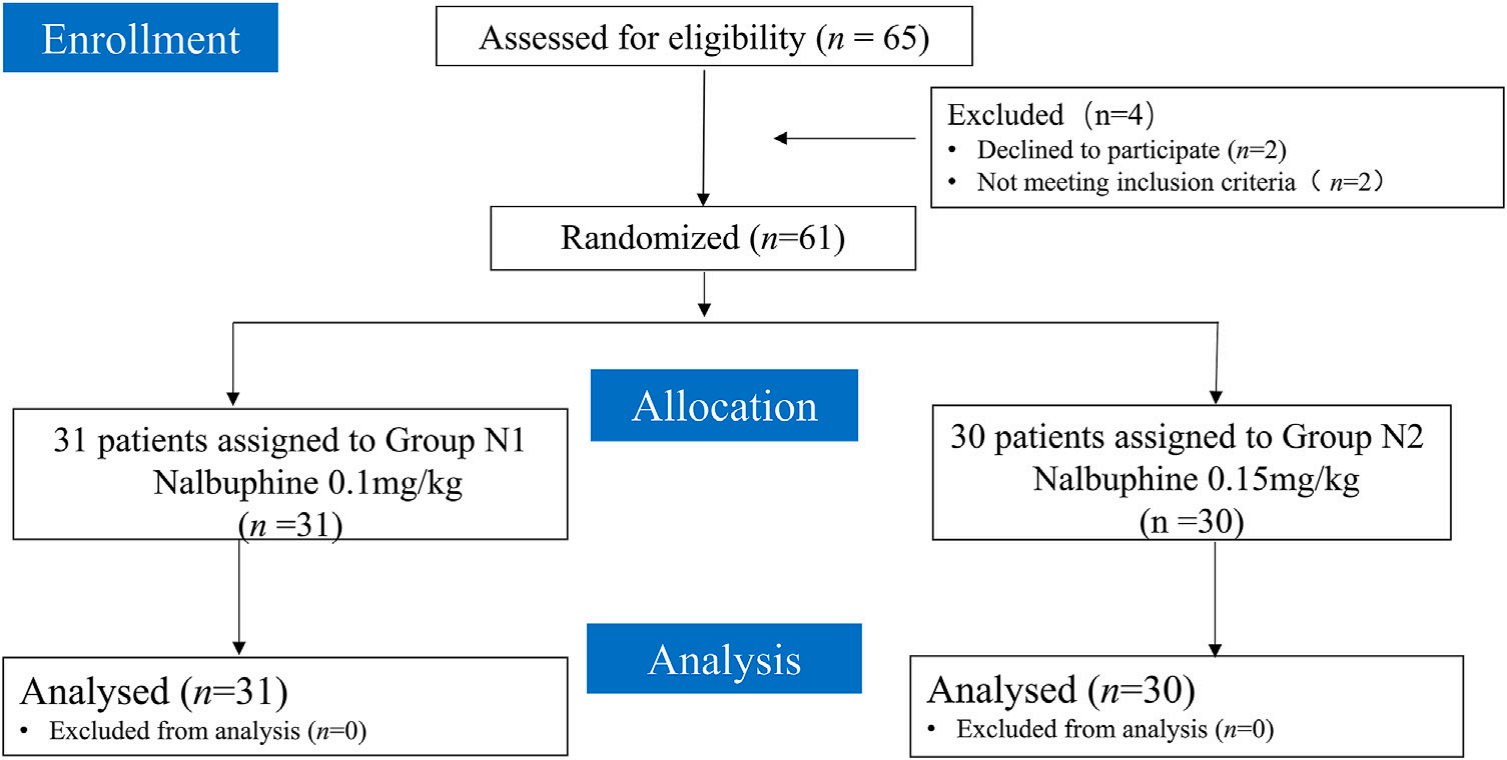
Flowchart of patient enrollment, allocation, and analysis.

From November 2021 to March 2022, sixty-five patients were assessed for eligibility, and four patients were excluded: two patients declined to participate, and two patients did not meet the inclusion criteria. When 31 patients were included in the N1 group and 30 patients in the N2 group, the 7 crossovers of each group occurred. Therefore, sixty-one patients were enrolled and allocated randomly to the N1 and N2 groups, and the data of 61 patients were finally analyzed (shown in Figure 1). The baseline data between the two groups showed no significant difference (Table 1).

**TABLE 1:** The general characteristics of patients in the two groups.

Values	Group N1 (*n* = 31)	Group N2 (*n* = 30)	*p* value
Age (years)	42.29 ± 11.30	43.30 ± 10.50	0.449
Sex (M/F)	10/21	10/20	0.572
Height (cm)	163 (160, 170)	162 (157.75, 168)	0.127
Weight (kg)	59.31 ± 7.19	58.45 ± 7.33	0.653
BMI (kg/m^2^)	21.22 (20.55, 22.83)	22.26 (21.05, 23.44)	0.132
ASA status			0.527
I	7 (22.58%)	6 (20%)	
II	24 (77.42%)	24 (80%)	

Values are expressed as the mean ± SD, median (IQR), or the number of patients and percent. M, male; F, female; BMI, body mass index; ASA, American Society of Anesthesiologists (ASA). No significant differences were found in these characteristics between the two groups. Group N1: 0.1 mg/kg nalbuphine; Group N2: 0.15 mg/kg nalbuphine; 3 min after nalbuphine administration, propofol was given to the two groups.

### The ED50/ED95 of propofol combined with different doses of nalbuphine

**FIGURE 2:**
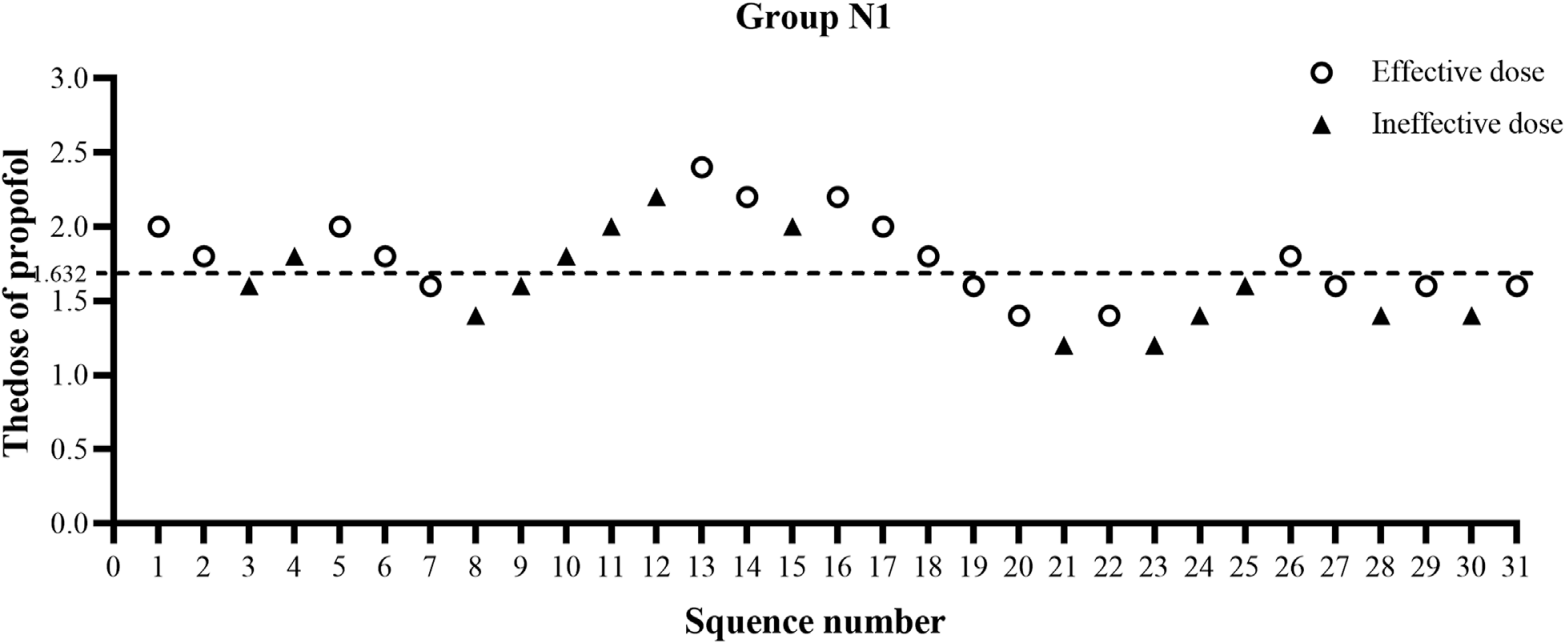
Sequential dose adjustment of propofol when combined with 0.1 mg/kg nalbuphine by the Dixon method in the N1 group. The open circle represents an effective dose; the filled triangle indicates an ineffective dose. The ED50 and ED95 were 1.632 mg/kg and 2.759 mg/kg, respectively, in the N1 group.

**FIGURE 3:**
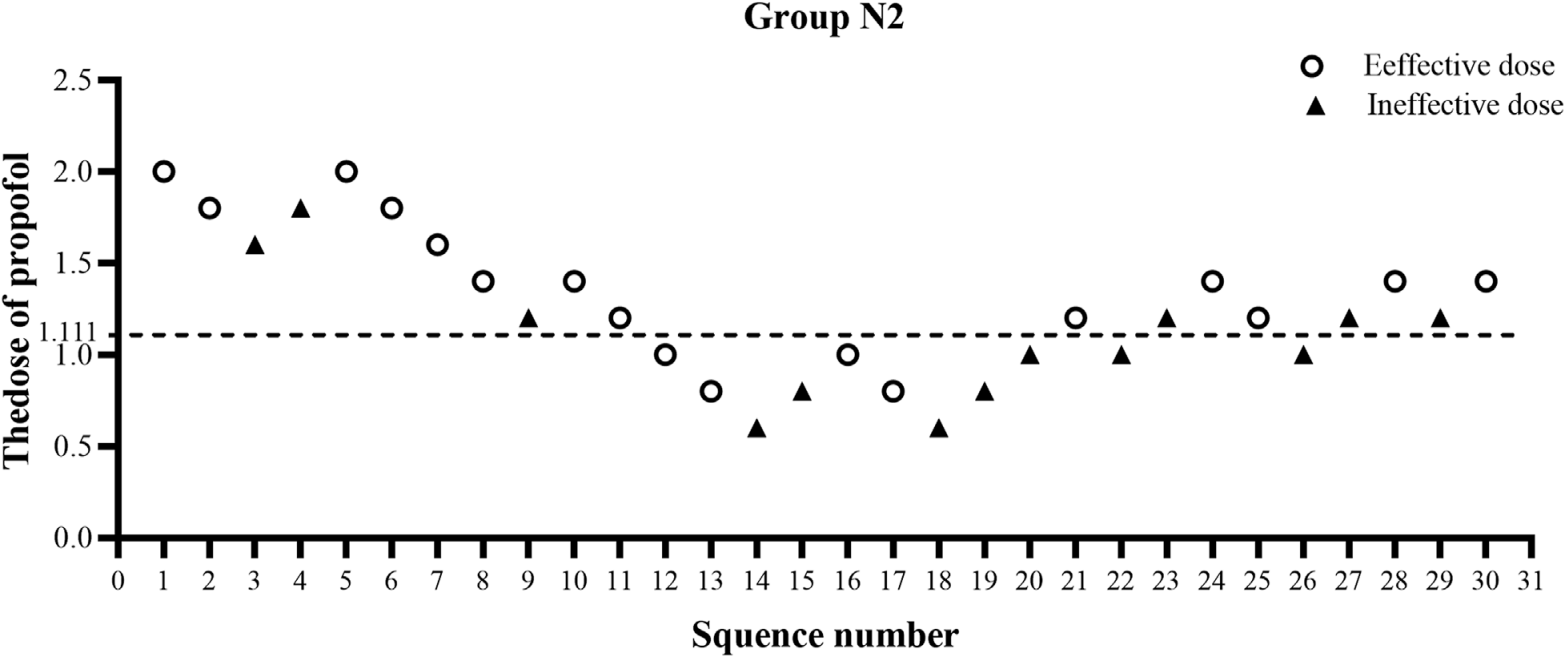
Sequential dose adjustment of propofol when combined with 0.15 mg/kg nalbuphine by the Dixon method in the N2 group. The open circle represents an effective dose; the filled triangle indicates an ineffective dose. The ED50 and ED95 were 1.111 mg/kg and 2.243 mg/kg, respectively, in the N2 group.

The ED50 of propofol in the N2 group was significantly lower than that observed in the N1 group (1.20 ± 0.38 vs. 1.66 ± 0.38 mg/kg, *p* < 0.01) (Table 2). The results of the ED50 of propofol in the two groups were similar using probit analysis, which were 1.632 mg/kg in the N1 group and 1.111 mg/kg in the N2 group. The ED95 values of propofol in the N1 and N2 groups were 2.759 mg/kg and 2.243 mg/kg, respectively. (shown in Figures 2, 3). The sequential doses of propofol combined with different doses of nalbuphine in gastroscopy are shown in Figures 2, 3.

**TABLE 2:** Comparison of perioperative outcomes and adverse events between the two groups.

Values	Group N1 (*n* = 31)	Group N2 (*n* = 30)	*p* value
Initial dose of propofol (mg)	102.35 ± 23.49	72.67 ± 23.54	0.000
Initial time of propofol (s)	64 (52, 77)	62 (53.75, 74)	0.874
Total dose of propofol (mg)	108 (93.60–134.80)	88.1 (62.85, 118.20)	0.002
ED50 of propofol	1.66 ± 0.38	1.20 ± 0.38	0.005
Duration of procedure (min)	4.66 ± 1.22	4.30 ± 1.90	0.390
Time of opening eyes (min)	6.43 (5.52, 7.93)	5.31 (3.99, 6.22)	0.014
Orientation recovery time	7.38 (6.25, 8.65)	6.00 (4.99, 7.36)	0.007
Stay of PACU (min)	15 (13.2, 17)	13 (12.10, 14.16)	0.018
VAS scores	0 (0, 1)	0 (0, 0)	0.030
Hypotension	11 (35.48%)	2 (6.67%)	0.011
Injection pain	9 (29.03%)	4 (13.33%)	0.118
Respiratory depression	8 (25.81%)	2 (6.67%)	0.081
PONV	1 (3.23%)	2 (6.67%)	0.612

Values are expressed as the mean ± SD, median (IQR), or the number of patients and percent. ED50, median effective dose; PACU, postanesthesia care unit; VAS, visual analog scale; PONV, postoperative nausea and/or vomiting.

### Comparison of intravenous anesthesia outcomes, procedure time and adverse effects

The initial time of propofol and the time of procedure did not differ between the two groups. Compared to the N1 group, the initial dose of propofol in the N2 group was significantly reduced (72.67 ± 23.54 vs. 102.35 ± 23.49 mg, *p* < 0.001). The total dose of propofol had similar results (88.1 [62.85, 118.20] vs. 108 [93.60, 134.80] mg, *p* < 0.01). The corresponding time of eye opening (5.31 [3.99, 6.22] vs. 6.43 [5.52, 7.93] min, *p* < 0.05), orientation recovery time (6.00 [4.99, 7.36] vs. 7.38 [6.25, 8.65] min, *p* < 0.01) and stay in the PACU (13 [12.10, 14.16] vs. 15 [13.20, 17.00] min, *p* < 0.05) were shorter in the N2 group than in the N1 group. There was a statistically significant difference in VAS score between the N1 and N2 groups (0 [0, 1] vs. 0 [0, 0]); *p* < 0.05). The incidence of injection pain of propofol and respiratory depression in the N2 group was slightly lower than that in the N1 group; however, there was no statistically significant difference between the two groups (*p* > 0.05). The incidence of nausea and vomiting between the two groups also showed no significant difference (*p* > 0.05). The results are all presented in Table 2.

### Comparison of hemodynamic and respiratory parameters at different time points

Table 3 shows that systolic blood pressure (SBP), diastolic blood pressure (DBP) and heart rate (HR) in the two groups were both significantly lower at T2 and T3 than at T1 (*p* < 0.05) (Table 3). The above indicators fluctuated more in the N1 group, and the incidence of hypotension in the N1 group was significantly higher than that in the N2 group (35.48% vs. 6.67%, *p* < 0.05). No difference was found in the respiratory rate values at different timepoints in each group or between the two groups (Table 3).

**TABLE 3:** Comparison of hemodynamic parameters and respiratory rate between the two groups at different time points.

Values	Time points	Group N1 (*n* = 31)	Group N2 (*n* = 30)
SBP (mmHg)	T1	131 ± 15.66	131.60 ± 15.49
	T2	113.71 ± 14.95*	121 ± 23.72*
	T3	108 ± 11.95*	112.60 ± 13.70*
	T4	112.35 ± 11.93	117.13 ± 11.71
DBP (mmHg)	T1	84.03 ± 13.80	86.20 ± 10.32
	T2	71.97 ± 13.20*	71.77 ± 11.28*
	T3	69.13 ± 10.30*	72.03 ± 11.47*
	T4	71.38 ± 12.65*	75.26 ± 11.93*
HR (bpm)	T1	84.35 ± 17.39	82.83 ± 11.31
	T2	79.38 ± 12.21*	75.23 ± 8.56*
	T3	74.74 ± 12.41*	75.63 ± 11.11*
	T4	80.13 ± 13.63	78.40 ± 11.06
RR (bpm)	T1	16.45 ± 3.37	15.77 ± 3.54
	T2	14.06 ± 3.07	14.03 ± 3.43
	T3	14.81 ± 3.36	14.2 ± 3.66
	T4	15.65 ± 3.13	16.00 ± 2.63

Values are expressed as the mean ± SD. SBP, systolic blood pressure; DBP, diastolic blood pressure; HR, heart rate; RR, respiratory rate. T1: before induction; T2: after induction; T3: end of gastroscopy; T4: opening eyes. Compared with T1, *p < 0.05.

## Discussion

Previous studies demonstrated that propofol is a safe and effective anesthetic for all endoscopy procedures, even for high-risk patients, including those with hepatic encephalopathy (Wang et al., 2013; Yin et al., 2019; Edelson et al., 2020; Dossa et al., 2021; Kang et al., 2021). However, propofol alone would likely lead to inadequate conditions for esophageal instrumentation (Cris D LaPierre et al., 2012). Therefore, opioids are added to improve tolerability and minimize adverse events associated with high-dose propofol in clinical practice. Several studies have shown that sufentanil, fentanyl, and ketamine reduce the ED50 of propofol and the incidence of propofol-related changes in hemodynamics for patients during different endoscopy procedures (Li et al., 2016; Hayes et al., 2018; Yu et al., 2019; Chen et al., 2021). Compared with μ receptor agonists, nalbuphine did not cause significant respiratory or cardiovascular depression, nausea and vomiting, or pruritus (Charuluxananan et al., 2001; Riviere, 2004). Another study in our group showed that 0.1 mg/kg nalbuphine can effectively inhibit pain caused by uterine contraction. Consequently, nalbuphine combined with propofol is superior to sufentanil combined with propofol for first-trimester abortion surgeries (Fang et al., 2022). Li and colleagues determined the safety and feasibility of nalbuphine combined with propofol for painless gastroscopy in adults (Li et al., 2021). Our study is the first randomized dose‒response study to evaluate the effect of different doses of nalbuphine on the ED50 of propofol during gastroscopy in adult patients.

Our study showed that 0.15 mg/kg nalbuphine leads to a significant reduction in the ED50 of propofol required to prevent cough or body movement during gastroscopy implantation in adult patients (BMI 18-24). The probit method demonstrated that the ED50 of propofol in combination with 0.15 mg/kg nalbuphine was 1.111 mg/kg and 1.632 mg/kg when combined with 0.1 mg/kg nalbuphine. The ED50 of propofol in the N2 group decreased by a dose of 0.521 mg/kg compared with that in the N1 group (an approximately 32% decrease). The ED95 of propofol in the N2 group also decreased by 18.7%. Thus, the effect of κ-mediated sedation depends on increasing the nalbuphine dose, which was consistent with previous studies (Chestnutt et al., 1987; Li et al., 2021). Additionally, the results of the initial dose and total dose of propofol were consistent with those between the two groups, which led to a shorter recovery time (6.00 [4.99, 7.36] vs. 7.38 [6.25, 8.65]) and a lower incidence of hypotension (35.48% vs. 6.67%). The incidence of hypotension in the N2 group decreased by more than 50% when our sample size was calculated at the time of 7 crossovers, which was less than the sample size we initially calculated. Consequently, this drug combination may be more suitable and safer for elderly patients and patients with cardiovascular disease. The higher turnover efficiency of painless gastroscopy (stay of PACU, 13 [12.10, 14.16] vs. 15 [13.20, 17.00]) reduced waiting time to improve patient satisfaction due to the large number of gastrointestinal endoscopies. However, 0.2 mg/kg nalbuphine combined with propofol was reported to not achieve more benefits than the 0.1 mg/kg nalbuphine group for hysteroscopy in Chen’s study (Chen et al., 2021), which was different from our results. This may be related to different types of procedural variables and gender.

A review reported that nalbuphine was equivalent to 0.8 to 0.9 times the analgesic effect of morphine and had a longer duration than morphine at equianalgesic doses, while there was a ceiling effect of respiratory depression and minimal effects on cardiovascular function (Heel, 1983). Therefore, nalbuphine is suitable for outpatient analgesia. As expected, two different doses of nalbuphine both provided effective analgesia for visceral pain and relieved patient discomfort caused by gastroscopy. Evidence showed a statistically significant difference in VAS scores between the two groups (0 [0, 1] vs. 0 [0, 0]), although it had no clinical significance. Our data of nalbuphine doses are in line with a recent prospective study, in which the ED50 and ED95 of nalbuphine combined with propofol for adult patients during gastroscopy were 0.078 and 0.162 mg/kg, respectively (Li et al., 2021). The incidence of propofol-induced Injection pain in the N1 group (29.03%) was similar to that in previous reports (27%) of our center (Wang et al., 2020), while that in the N2 group (13.33%) was lower. In our study, the incidence of respiratory depression (<SpO_2_ 95%) was 25.81% in the N1 group and 6.67% in the N2 group. No severe hypoxia occurred in either group, which was lower than the rate reported in a previous study (8.4%), while the prevalence of subclinical hypoxia in the N2 group was lower than that reported in a previous study (16.3%) (Lin et al., 2019). The two groups had similar fluctuations in SBP, DBP, and HR at T1, T2, T3 and T4. SBP, DBP and HR at T2 and T3 significantly decreased compared with those at T1. The hemodynamic decline was slightly greater in the N1 group. This should be attributed to the larger propofol dose. The respiration rate (RR) in the two groups did not differ much at T1, T2, T3 and T4. In conclusion, nalbuphine can effectively suppress visceral pain during gastroscopy with stable hemodynamics and mild respiratory depression.

Our study has some limitations. First, we only evaluated the ED50 of patients with BMI 18-24, excluding overweight and obese patients. Second, all of the participants were healthy adults with ASA I or II, excluding elderly patients or ASA III or higher patients. Accordingly, the recommended dose of nalbuphine combined with propofol in our study cannot be extended to these populations. Future studies should examine the dosages of nalbuphine combined with propofol among other populations.

## Conclusion

In summary, the current study indicates that the ED50 values of propofol combined with nalbuphine are 1.632 (0.1 mg/kg of nalbuphine) and 1.111 mg/kg (0.15 mg/kg of nalbuphine), respectively. Treatment with 0.15 mg/kg nalbuphine led to a significant reduction in the ED50 of propofol, reduced the incidence of hypotension and shortened the recovery time. Therefore, nalbuphine (0.15 mg/kg) is a safe option for enhancing recovery after painless gastroscopy.

## Data Availability

The raw data supporting the conclusions of this article will be made available by the authors, without undue reservation.
